# Parental perceptions of a novel subsidy program to address the financial burden of milk allergy: a qualitative study

**DOI:** 10.1186/s13223-023-00828-w

**Published:** 2023-07-29

**Authors:** Manvir Bhamra, Zoe Harbottle, Michael A Golding, Moshe Ben-Shoshan, Leslie E Roos, Elissa M Abrams, Sara J Penner, Jo-Anne St-Vincent, Jennifer LP Protudjer

**Affiliations:** 1 Department of Food and Human Nutritional Sciences, University of Manitoba, Winnipeg, MB Canada; 2 The Children’s Hospital Research Institute of Manitoba, Winnipeg, MB Canada; 3 Department of Pediatrics and Child Health, University of Manitoba, Winnipeg, MB Canada; 4 Department of Allergy and Immunology, McGill University, Montreal, QC Canada; 5 Department of Psychology, University of Manitoba, Winnipeg, MB Canada; 6 Department of Business and Administration, University of Winnipeg, Winnipeg, MB Canada; 7 Children’s Allergy and Asthma Education Centre, Winnipeg, MB Canada; 8 George and Fay Yee Centre for Healthcare Innovation, Winnipeg, MB Canada; 9 Institute of Environmental Medicine, Karolinska Institutet, Stockholm, Sweden

**Keywords:** Costs, Food allergy, Intervention

## Abstract

**Background:**

Approximately 6–7% of Canadian children have food allergy. These families face substantial burdens due to the additional costs incurred purchasing allergy-friendly products necessary for management compared to families without food allergies. In the year prior to the COVID-19 pandemic, these costs were equivalent to an average of $200 monthly compared to families without food allergy. As food prices continue to rise, rates of food insecurity also increase, disproportionately affecting households with food allergy who have limited choices at food banks.

**Methods:**

Families living or working in Winnipeg, Canada with an annual net income of about $70,000 or less the year prior to recruitment and a child under the age of 6 years old with a physician diagnosed milk allergy were recruited between January and February 2022. Participating families received bi-weekly home deliveries for six months, from March to August 2022, of subsidy kits containing ~$50 worth of milk allergy-friendly products. Semi-structured interviews, completed ± 2 weeks from the final delivery, were audio-recorded, transcribed verbatim, and analyzed thematically.

**Results:**

Eight interviews, averaging 32 min (range 22–54 min), were completed with mothers from all different families. On average, mothers were 29.88 ± 4.39 years old and children were 2.06 ± 1.32 years old. All children reported allergies in addition to milk. Based on the data from these interviews, we identified 3 themes: food allergy causes substantial burden for families, “I have to get his allergy-friendly food first before getting to my basic needs”, and perceived emotional and financial benefits of a milk allergy-friendly food subsidy program.

**Conclusions:**

This study, along with previous research, suggests that there is a need for assistance for families managing milk allergies. It also provides important information to inform development of programs which can address these financial challenges. Our in-kind food subsidy was perceived as having a positive impact on food costs and stress associated with food allergy management, however, parents identified a need for more variety in the food packages. Future programs should strive to incorporate a greater variety of products to address this limitation.

## Introduction

An estimated 6–7% of Canadian children have food allergy [1], for whom dietary avoidance is essential to prevent a potentially severe allergic reaction [2]. While the prevalence of probable food allergy does not appear to differ between children in low- vs. high-income Canadian households [3], it is plausible to speculate that the food allergy management does differ between income groups. Indeed, prior to the COVID-19 pandemic, Canadian households managing food allergy reported significantly greater food costs, of an average of $200 monthly, compared to households not managing food allergy [4]. Also prior to the COVID-19 pandemic, 14% of mothers reported career limitations due to their child’s food allergy [5], perhaps, in part, attributable to the substantial practical and social burdens, and unpredictable risks of exposure associated with food allergy management [6].

Yet, over the course of the COVID-19 pandemic, the burden of food allergy has shifted. In the early months of the pandemic, households managing food allergy reported further excess food costs, coupled with difficulties procuring allergy-safe foods, which, in turn, contributed to indirect (time) costs [7]. Despite these increased costs, COVID-related restrictions, including stay-at-home guidance, contributed to decreased food allergy-specific anxiety [8, 9].

As the COVID-19 pandemic persisted, food prices have increased by as much as 8% annually [10–13]. Concurrently, supply chain issues have limited product availability [14]. These events have culminated in high rates of food insecurity [15] and food bank usage [16] amongst the general population, and which are likely to continue through the COVID-19 recovery. Yet, food insecurity disproportionately affects households with food allergy [17–19], for whom there are also more limited choices at food banks [20–22].

The COVID-19 pandemic has also normalised home delivery of grocery purchases, from 19% prior to the pandemic, to 49% after the first year of the pandemic [23]. Changes in purchasing habits created novel opportunities to support households [24–26]. However, to our knowledge, home delivery of cost-free allergen-friendly foods to support households managing food allergy has not previously been described.

As the decision of what to eat is intensely personal, and motivated by many factors, including but not limited to culture, medical dietary restrictions and household finances, the collection of multiple types of data are critical to glean a complete understanding of the impact of food home delivery programs to support households managing food allergy. In the present study, we aimed to qualitatively describe how Winnipeg-based families with a child under the age of 6 years with a physician diagnosis of milk allergy perceived a bi-weekly home delivery intervention, which aimed to off-set the added costs of an allergen-friendly diet.

## Methods

The present study was part of a novel, mixed methods intervention to support lower income households while concurrently managing their child’s milk allergy. To be eligible for this study, families had to have an annual household income of about $70,000 or less the year prior to recruitment, have a child under the age of 6 years with a physician diagnosis of milk allergy, and live or work in Winnipeg, Canada. Families were recruited via a database maintained by the principal investigator (JP), via social media channels and word-of-mouth. Families who did not have a physician letter confirming diagnosis were asked to obtain a letter to this effect, for which they were reimbursed through research funds supporting this project. As the intervention consisted of an industry-supported subsidy of products that contained peas, coconut and beans, families were ineligible to participate if they reported allergies to one or more of these foods. Interested and eligible families were provided a study information letter, and were encouraged to ask questions prior to consenting. Upon consent, families also provided a detailed list of other dietary restrictions; these included consideration of allergies to foods in addition to milk, as well as other medical dietary restrictions, and, or restrictions of any other kind. All participants were recruited between January and February 2022.

Starting in March 2022, for six months, participating families received a food subsidy every two weeks, which members of our research team, working in pairs, delivered to families’ homes. Food subsidies were valued at ~$50 (range $34-$73) and contained coupons for either free products or $1 off Daiya products as well as 6–11 allergen-friendly food products including a variety of dairy-free macaroni and cheese, dairy-free cheese (block, shredded, or slices), dairy-free cream cheese, and dairy-free salad dressing. The composition of each subsidy package was dependent on the products provided by our industry partner. As COVID-19 restrictions were in place at the start of the study, home delivery was considered to be preferable to central distribution. One hour prior to each delivery, team members telephoned the designated adult for each family, to ensure that an adult household member would be home to accept delivery of the foods, at a mutually agreed upon location or at the participant’s home. All deliveries were contactless, and the research team was required to wear a mask and maintain a distance of six feet or 2 metres from participants at all times.

Data collection for this study included a range of quantitative questionnaires (collected at baseline, midpoint [i.e. 3 months] and endpoint, as well as qualitative interviews (see Table 1 for select questions) with parents which were completed +/- 2 weeks from the final subsidy delivery. These interviews are the focus of the present study.

**Table 1:** Select questions from the interview guide

What kinds of changes have you had to make because of the food allergy?
What does your family think about the cost of these allergy-friendly foods?
You participated in a six-month study with an allergy-friendly food subsidy. What did you think of the program?

Demographic characteristics of parents participating in this interview study were extracted from baseline survey data, and analysed descriptively (n/N, %, mean ± standard deviation [SD]), using Stata Version 17.0, College Station, TX. Each interview was conducted by telephone, audio-recorded and transcribed verbatim. Transcripts were thematically analyzed independently, but concurrently by [27] two research assistants (MB, ZH) under the mentorship of two authors (MG, JP) who have expertise in qualitative methods. In brief, the analysis involved two stages. First, analysts read the transcripts for surface descriptive content, and organized like-with-like ideas. Second, transcripts were re-read for latent content to better understand participants’ meaning. As part of this process analysts looked for contradictory or confirmatory statements. Each analyst independently generated and systematically applied themes across all transcripts. Semantic validity checks ensure different words and phrases within a category have similar meanings [27], so meaning is “not lost in translation.” Constructs within this study were deemed saturated (akin to statistical significance in quantitative work) when no new or additional constructs were identified, consensus was reached on all overarching themes, and no alternative explanations were found with subsequent interviews.

This study was approved by the University of Manitoba Health Research Ethics Board (HS25168 [H2021:340]). The industry partner provided product in kind and had no influence on the presentation of the findings reported herein.

## Results

A total of eight parents, all mothers from different families, participated in semi-structured interviews, averaging 32 min (range 22–54 min). On average, mothers were 29.88 ± 4.39 years old, with few (n = 2) having a food allergy themselves. Children were, on average, 2.06 ± 1.32 years old, with an even distribution of boys and girls (n = 4 each). All children had a milk allergy and reported additional allergies (Table 2). Based on data from these interviews, we identified three themes (Table 3), each of which is described below.

**Table 2:** Participant Characteristics (N = 8 mothers)

Variable	%	*n*	Mean	SD
**Household characteristics**				
Number of children			2.13	1.46
Number of adults in the house			2.25	0.71
Married, common law	87.50%	7		
Annual household income (N = 7)				
≤$24,000	57.14%	4		
$40,001-$70,000	42.86%	3		
**Child with food allergy characteristics**				
Number of food allergies			2.88	1.13
Additional food allergies*				
Egg	50%	4		
Peanut	50%	4		
Soy	37.5%	3		
Shellfish	37.5%	3		
Fish	12.5%	1		
Tree nuts	12.5%	1		
Sesame seeds	12.5%	1		
Wheat	12.5%	1		

*Not mutually exclusive

**Table 3:** Summary of themes and supporting quotations

Theme	Theme Description	Supporting Quotations
Food allergy causes substantial burden for families	Having a child with food allergy can cause many challenges for families. This was demonstrated by the additional effort required for management.	“Just having to read every single label was a huge eye opener, having to pretty much switch most of my recipes for dinners … it was a big change for us.” “I have to make everything on my own for him and then he also gets the jealous factor of what everyone else is eating, so [the childcare has] a 3-week rotating meal plan that I try to follow so he does not get jealous of what the other kids are having.”
“I have to get his allergy-friendly food first before getting to my basic needs”	Parents described sacrifices made to meet their child’s dietary needs. Including putting the child’s needs above their own, and avoidance of the child’s allergen by the entire family for risk of cross contamination.	“[I] have to get his [food] first before getting to my basic needs … and sometimes I have to put stuff on credit versus putting things back if I absolutely need them.” “I had to have a very strict budget and sometimes I would have to put things back for myself that I would need just to accommodate for the things he needed for his allergies.” “For myself personally, my consumption of it [child’s allergen] changed significantly because I will barely have it when he is around just to not cross contaminate anything.”
Perceived emotional and financial benefits of an allergy-friendly food subsidy program	Parents spoke of this food subsidy program having many positive benefits, emotionally and financially, especially for those with recent diagnosis.	“He was able to try these new things [dairy-free products] without going into our budget of our regular spending.” “We spent less on these particular items, because I would never have to go to the store and buy dairy-free cheese, so it definitely helped for sure without cutting down.” “It was really getting to try all the different things, see the range of products that we did not necessarily even know were out there.”

### Theme 1: Food allergy causes substantial burden for families

Having a child with food allergy was perceived as placing substantial burden on the entire family, which was, in part, attributable to additional efforts required to accommodate their child’s food allergies. Parents described how these efforts were particularly burdensome directly after diagnosis. Learning to read labels for allergens, the additional time necessary to grocery shop, finding appropriate products, and the knowledge required to manage food allergy are all examples of the additional effort required post-diagnosis:

*Just having to read every single label was a huge eye opener, having to pretty much switch most of my recipes for dinners … it was a big change for us*.

Another parent succinctly summarised this as “*so difficult*”.

While some of these burdens improved with time and knowledge, some persisted beyond the initial diagnosis. In particular, many families reported having to alter meals to make them “*everybody friendly*”, or even make multiple meals: one for the child with food allergy and one for the rest of the family.

Some parents, largely those with children with many food allergies, reported that their child’s allergy was not accommodated by their school or child care centre, thereby creating a situation in which parents were required to prepare additional meals to send with the child to school or child care. In cases where children could not be accommodated, substantial efforts were made by parents to prepare the same snacks and meals that were provided to other students by the school/child care. Parents explained that this was done to prevent their child from feeling excluded or that they differed from their peers. As one parent said:

*I have to make everything on my own for him and then he also gets the jealous factor of what everyone else is eating, so [the childcare has] a 3-week rotating meal plan that I try to follow so he does not get jealous of what the other kids are having*.

### Theme 2: “I have to get his allergy-friendly food first before getting to my basic needs ”

Parents described challenges in ensuring their child had safe and appropriate food. These challenges ranged from additional food costs related to the notable difference in price between allergy-friendly and non-allergy-friendly products. Parents spoke about the costs of allergy-friendly food products, as being “*absolutely ridiculous*.”. In some cases, the high costs of allergen-friendly foods resulted in parents prioritizing the dietary needs of their child over their own. One parent noted,

*[I] have to get his [food] first before getting to my basic needs … and sometimes I have to put stuff on credit versus putting things back if I absolutely need them*.

Beyond cost-related compromises, parents reported that they and other family members consumed less of or avoided their child’s allergen altogether in order to reduce the risk of cross-contamination. For example, one participant explained that:

*For myself personally, my consumption of it [child’s allergen] changed significantly because I will barely have it when he is around just to not cross contaminate anything.*

While this practice helped to ensure the safety of the child with food allergy, it limited the range of foods available to the household, which made it difficult to find meals and snacks that satisfied the entire family.

### Theme 3: perceived emotional and financial benefits of a milk allergy-friendly food subsidy program

Parents spoke of the food subsidy program in largely positive terms and reported several benefits including cost savings. Dairy-free cheese-like products formed a primary component of the food packages; which is a higher price-point grocery item. Families perceived the provision of such foods as a luxury item. But, the provision of such items as part of a subsidy created an opportunity in which families were able to try new food items, without any financial impact on their food budget.

*We spent less on these particular items, because I would never have to go to the store and buy dairy-free cheese, so it definitely helped for sure without cutting down*.

Parents also noted a reduction in stress, as their worries related to the costs of purchasing allergy-friendly foods were perceived to be lessened. In turn, parents described they no longer needed to ration expensive allergen-friendly products, like dairy-free cheese, and instead felt free to use as much product as they desired when preparing meals. Indeed, the current program provided an opportunity for families to try a variety of novel products by removing any financial constraints and risk. As one parent commented,

“*I feel like this is a good way to kind of introduce yourself to a different variety of stuff and like with the coupons* [free product coupons], *we get to have a little bit of flexibility to try different things, which is nice*.”

For families whose children had been newly diagnosed with milk allergy, the subsidy had an additional benefit. These families were still learning to navigate food purchasing, and thus were not familiar with the range of available products. In addition to providing exposure to new products, participants also reported appreciating the “in-kind” nature of the program, which provided them with tangible food products rather than cash benefits, as it ensured that benefits went directly towards managing their child’s food allergy

*It was really getting to try all the different things, see the range of products that we did not necessarily even know were out there*.

Given these perceived benefits, it is not surprising that there was overwhelming support for the continuation of a subsidy program: *“It would be extremely helpful if [the subsidy] did continue.”* That being said, the program was not without limitations. Most notably, families indicated that it would be preferable if the food packages contained a greater variety of products beyond dairy-free cheese and prepared goods. Because the packages were perceived to lack variety, some families struggled to consume all of the items before they received the next package: “*Because of the amount of cheese there was it [the frequency of deliveries] was almost too often for us*.”

## Discussion

In this qualitative study of parents’ perceptions of a novel subsidy program that aims to address the financial burden of milk allergy, three primary themes were identified. Parents described how childhood food allergy imposes a burden on the entire family, with some parents reporting the need to prioritize their child’s dietary needs above their own. However, the in-kind subsidy program was perceived to reduce this burden by decreasing food costs and stress. These perceived benefits led to parents expressing overwhelming support for the continuation of the program.

Consistent with previous studies, parents described how managing their child’s food allergy placed substantial financial and practical burdens upon their entire family particularly in cases where resources were already limited [4, 18, 28, 29]. As a result of the financial strain imposed by food allergy, some parents indicated that they were required to prioritize their child’s dietary needs over their own by purchasing less food for themselves. To date, this phenomenon has not been described in the context of food allergy; however, research on food insecurity has found that mothers prioritize their child’s nutritional needs over their own if they do not have the financial resources to support both [30]. More than one in four children in Manitoba live in poverty leading to a reliance on food banks [31], with an alarming 33.1% of Canadian food bank users being children [16]. The additional costs incurred as a part of food allergy management can further exacerbate food insecurity and increase food bank use [17–19], which for those with food allergy, may offer limited choices [20–22].

In addition to financial costs, parents indicated how accommodating their child’s food allergy requires additional practical effort when shopping and preparing food. This finding is concordant with previous qualitative work, which has found that the practical aspects required to manage a childhood food allergy, including finding appropriate allergen-friendly food, label reading, and preparing additional meals for the child with food allergy, are both time-consuming and burdensome [32–34]. While the current study helped to corroborate previous findings on the practical burden of allergy, it also provided novel evidence these burdens are heightened when a child’s food allergy is not accommodated by their school/childcare centre as it means parents are responsible for preparing a lunch for their child themselves.

Parents had positive experiences participating in the food subsidy program, which resulted in cost savings and reduced stress related to purchasing allergy-friendly foods. As food packages consisted of higher price point allergy-friendly items such as dairy-free cheese-like products, this allowed families to try new products while alleviating worries related to cost and their child’s enjoyment of these products. This aspect of the in-kind subsidy program was particularly beneficial for newly diagnosed families as they were not necessarily aware of the range of allergy-friendly products available. Previous research suggests that families, following the initial diagnosis, often struggle with the practical aspects involved in managing a food allergy. While these struggles have been discussed in regards to avoiding allergens, label reading, and advocating on their child’s behalf, findings from the current study also suggest that parents are often unaware of the full range of allergen-friendly products available [34, 35].

Both the current findings and previous research suggest that more financial support is needed for lower-income Canadians with food allergy [4, 28, 29]. However, more research is needed to not only understand the best manner for providing this support, but to also evaluate its feasibility and political acceptability, if the support is taxpayer funded. Unfortunately, the need for support is likely greater now than it has ever been as the COVID-19 pandemic has resulted in what has been described as the “largest increase in grocery prices in Canadian history” [36]. While all families are likely feeling the effects of the increase in food prices, those managing food allergy appear to be especially burdened. In the year prior to the COVID-19 pandemic, it was reported that families managing food allergy spent an average of $200 more per month on food than families without food allergy [4]. These costs increased in the beginning of the pandemic when families managing food allergy reported further increases of $99-$213 monthly in food costs alone, varying in association with household income [7]. Unfortunately, food prices are expected to increase by 5 to 7% over the course of 2023 [13], which is likely to place lower income households with food allergy under increasing strain.

Despite the high costs of food allergy, there are currently no government-funded programs designed to help offset the costs of food allergy in Canada. Individuals with celiac disease are eligible to claim the additional cost of gluten-free food on their federal income tax, but the same benefits are not available to individuals with food allergy. While any form of additional support would likely be appreciated, parents in the current study indicated a preference for in-kind food subsidies, rather than a tax credit, as the program provided them with exposure to a wide variety of products and ensured that the funds were used strictly for milk allergy-friendly food products. The in-kind nature of the program was not without limitations, however, as many participants cited a lack of variety in the food packages as a shortfall of the program. While a lack of choice is a common criticism of in-kind benefits (i.e., goods or services rather than cash), this limitation could be reduced by including a greater variety of food products or through greater use of coupons that could be used to purchase a variety of products [37, 38].

While the current study provided evidence of a potentially positive impact of a milk allergy benefit program, it was not without limitations. One of the primary limitations is the small sample size of the study, which consisted solely of women. This lack of gender diversity may limit the transferability of the findings to a broader population. Therefore, future studies should seek to include a more diverse participant pool. The study also did not attempt to address the sustainability of the program. It is important to consider the financial feasibility of continuing the program, as well as the implementation and scalability. Future studies should consider exploring ways to make the food-subsidy program more sustainable over the long-term. This may involve partnerships with government agencies, non-profit organizations, or private sector entities to support funding and ensure continuity. As well, due to the nature of qualitative interviews, there is the potential for courtesy bias. As parents appreciated the products provided, they may be inclined to emphasize the positive aspects of the study to not offend the researcher [39, 40].

A strength of this study was that it provided unique insight into the lived experiences of families with food allergies, which is an important and often overlooked perspective. This was achieved through qualitative interviews, in which participants were able to freely discuss both their experiences with managing food allergies and the impact of the subsidy program. Additionally, this is the first known project to investigate the implementation of a program aimed at reducing costs associated with milk allergy management, highlighting the innovative nature of the research.

Mothers in the present study reported consuming less of, or avoiding their child’s allergen entirely to reduce the risk of cross-contamination. In this study, all children had physician-diagnosed milk allergy. Milk is nearly ubiquitous in most food supply chains, thus increasing the likelihood of exposure to milk for other children in the household. However, this restricted/avoidance behaviour, if extrapolated to other allergens, such as peanuts or tree nuts, which may be more easily avoided, raises the possibility that other children in the household may not be exposed to these foods from an early age, on a regular basis. Yet, mounting evidence supports early and regular introduction, by ages 4–6 months, is protective against food allergy [41–43]. To this end, the Canadian Society of Allergy and Immunology supports early and regular ingestion of common allergens, for a duration of 5 years, which appears to be adequate to establish and maintain tolerance [44].

In conclusion, the current study provides valuable information that can be used to inform the development of programs aimed at addressing the financial challenges faced by families with milk allergies. In the current study, our in-kind food subsidy program was perceived as having a positive impact on food costs and stress; however, participants did indicate a need for more variety in the food packages. Future programs aimed at addressing the financial burden of milk allergy should strive to address this limitation, while also finding innovative ways to promote sustainability.

## Data Availability

Owing to the highly personal nature of qualitative data, requests for data will be carefully vetted by a minimum of three authors.

## Abbreviations

SD: Standard deviation
